# The microbiome in precision medicine: the way forward

**DOI:** 10.1186/s13073-018-0525-6

**Published:** 2018-02-22

**Authors:** Joseph F. Petrosino

**Affiliations:** Alkek Center for Metagenomics and Microbiome Research, Department of Molecular Virology and Microbiology, Baylor College of Medicine, Houston, TX 77030 USA

## Abstract

Novel associations between the human microbiome and health and disease are routinely emerging, and important host–microbiome interactions are targets for new diagnostics and therapeutics. Understanding how broadly host–microbe associations are maintained across populations is revealing individualized host–microbiome phenotypes that can be integrated with other ‘omics’ data sets to enhance precision medicine.

## The microbiome is a key component of precision medicine

The prospect of individualized medical therapies that are customized for maximal efficacy has accelerated therapeutic and diagnostic discoveries that incorporate comprehensive patient profiles, including demographics and family history; traditional laboratory data; and next-generation ‘omics’ data, such as human genomic, metabolomic, and proteomic readouts. As a result, cohort-level analyses involved in biomarker discovery are becoming more sophisticated to include complex biostatistics, machine learning, and artificial intelligence algorithms to parse out associations with disease that will only increase the therapeutic utility of these supplemental data types. New programs, such as the National Institutes of Health (NIH) All of Us Initiative, have been created to build and analyze the types of data that are thought to be able to bring refinement to precision medicine biomarker discovery.

The human microbiome, dynamic communities of microbes that colonize the body, provide a variety of metabolic functions and molecular signals to maintain proper health. It also provides colonization resistance from dangerous pathogens such as *Clostridium difficile.* The ever-increasing understanding of how the microbiome affects health and disease clearly suggests that human microbiome data should be included in precision medicine approaches. The implementation of next-generation sequencing strategies as a means to profile the entire microbial composition at a given body site has accelerated the study of the networks of microbes, the genomic content of which outnumbers the host-encoded functions in a given individual by at least an order of magnitude. The field of microbiome research has paralleled that of human genomics/genetics in that advancements in genomic sequencing platforms have ushered in an era of excitement by unlocking the genetic code, both human and microbial, to reveal treatment opportunities for myriad diseases. After initial milestones were completed (for example, the complete determination of the human genome in 2003; the NIH Human Microbiome Project, phase 1, which ended in 2013; the European Union MetaHit Project, phase 1, which ended in 2012; among other initiatives) expectations for new therapies ran high, and with breakthroughs being slow to emerge, some skepticism arose, but funding agencies and institutions have stayed the course and doubled-down on these important areas of research. The incorporation of human genomic information into patient care and diagnosis is now routine. The identification of polymorphisms in the host genome that result in disease susceptibility (or protection) and that affect responsiveness to treatment lies at the heart of the precision medicine movement. The question is: can human microbiome knowledge be utilized in the same manner? And, if so, how?

## Establishing microbiome-encoded disease phenotypes

Human genomics benefits from a relatively static set of genes (compared with microbial genomes) with polymorphisms that can be traced and associated to disease using genome-wide association studies (GWAS) for complex diseases and using case-parent trios for Mendelian disease. Microbiome associations with disease may be derived from function(s) encoded by one species or strain [1], akin to Mendelian or single gene disorders that can be treated with supplementation of a metabolite or gene product. Meanwhile, other phenotypes may mirror complex human disease, where many gene deficiencies are involved, and require the contributions of multiple bacterial species [2], or overall diversity, to restore health. Numerous studies have revealed microbiome–disease associations; however, determining causality is often a challenge. Linking true microbiome associations with disease is complex and usually requires longitudinal sampling and rigorous informatic approaches to accurately assess changes in the fluctuating microbiome over time. Furthermore, observations of taxonomic associations with certain disease states may not always agree from study to study and is likely to reflect microbiome heterogeneity that is influenced by host genetics and environmental exposures that factor in the establishment and maintenance of the microbiome over the course of life.

Despite these challenges, microbiome-wide association studies (MWAS) and other approaches are revealing microbiome contributions to human health and disease [3]. One overarching conclusion from these efforts is that many diseases are impacted by the microbiome’s capacity to modulate the immune system, specifically in its ability to influence levels of inflammation in the gut, as well as systemically, with some mechanisms revealed in animal models and/or translational studies [4]. Once causality is established, implementing this knowledge to improve disease outcomes through new diagnostics and therapeutics becomes the imperative, including understanding how widespread given associations are across populations of individuals. This latter component is central to the development of early microbiome-based precision medicine diagnostics and therapeutics.

Examples in recent papers have revealed ways in which the microbiome may play a role in personalized medicine through the immune response. Three articles published in January 2018 illustrate how differential responses to immune checkpoint blockade treatments targeting programmed cell death protein 1 (PD-1)/ programmed cell death 1 ligand 1 (PD-L1) are associated with the patient gut microbiome profile [5–7]. Two of these studies examined the gut microbiome in patients with metastatic melanoma being treated with PD-L1 blockade therapy. Interestingly, a different set of bacterial taxa were associated with successful outcomes [5, 6]. Gopalakrishnan et al. revealed that the relative levels of the bacterial genus *Faecalibacterium* were elevated in patients who responded better to PD-1 checkpoint therapy, while the levels of bacteria belonging to the order Bacteroidales were increased in fecal samples from patients who responded poorly to treatment [5]. Meanwhile, Matson et al. found levels of eight species to be elevated in responders, including two belonging to the *Bifidobacterium* genus, while levels of *Ruminococcus obeum* (recently reclassified as *Blautia obeum*) and *Roseburia intestinalis* were increased in non-responders [6]. These different taxonomic associations with outcome may be partly due to discordance in patient demographics, host genetics and/or environmental exposures from different geographical regions, among other possibilities. Subsequent experiments have demonstrated that these taxa are improving the immune response to tumors through T-cell infiltration and/or activation [6]. Dissecting why different bacterial taxa appear to influence the same treatment of the same disease in different individuals could lead to diagnostics that better predict treatment success and/or that provide additional treatment options to enhance the chances of success.

## Precision editing of the microbiome

Once disease associations are made, another important component required to incorporate the microbiome in precision medicine is the development of methods to modify the microbiome for the benefit of the patient. In a provocative study recently published by Zhu et al., the authors demonstrated how precision editing of the gut microbiota may be used as a treatment for gastrointestinal inflammatory disease [8]. The authors had previously identified Enterobacteriaceae family expansion and overrepresentation of molybdenum-cofactor-dependent metabolic pathways in a model of chemically induced colitis. Molybdenum-cofactor-dependent pathways are essential for the overgrowth of Enterobacteriaceae in the inflamed gut [9], and Zhu et al. demonstrated the targeted inhibition of these pathways by oral administration of tungstate, as tungsten can replace molybdenum in the molybdopterin cofactor. The resulting restriction of Enterobacteriaceae growth restored the microbial diversity to a normal state. Furthermore, colitis-associated inflammation was reduced in the tungstate-treated animals by up to 90%.

In the next step toward translating this treatment to humans, the authors took gut microbiota from a subset of patients with inflammatory bowel disease (IBD) and transferred these communities into germ-free mice. When inducing colitis, animals receiving tungstate showed decreased Enterobacteriaceae expansion and associated markers of inflammation, thereby demonstrating that this treatment, or other means of inhibiting molybdenum-cofactor-dependent pathways in bacteria, may be an effective means of controlling inflammation in patients with IBD [8]. This targeted approach to managing dysbiosis-associated inflammation, without affecting beneficial microbes, represents a significant advancement toward precision medicine approaches to manipulating the microbiome, particularly with respect to targeting or repressing the immune response. And, as more microbial pathways that cause or exacerbate diseases are identified, the more potential therapeutic and diagnostic targets clinicians will be able to exploit to treat these conditions.

## The route to precision medicine

Studies such as these are highlighting the future of microbiome-based precision medicine treatments and diagnostics. Patients entering immunotherapy treatment for cancer or other diseases may have their gut microbiota profiled to determine whether the immune system is in an optimal state for treatment, and patients in need may receive a microbiome-modifying pre−/co-treatment to edit the microbiome so that its constituents can best prepare the patient for optimal treatment. In addition to stratifying patients for immunotherapy treatment, the microbiome has the potential to stratify patients for a variety of other immune or inflammatory-related diseases. This extends to clinical trials; for example, the microbiome in responders versus non-responders from an early phase drug trial could be used to inform patient selection and subsequent prescription of the drug under investigation. In addition to responsiveness to treatment, the microbiome has been shown to affect the metabolism of certain drugs [10] and thus should be considered in patient pharmacogenomic profiles.

**Fig. 1:**
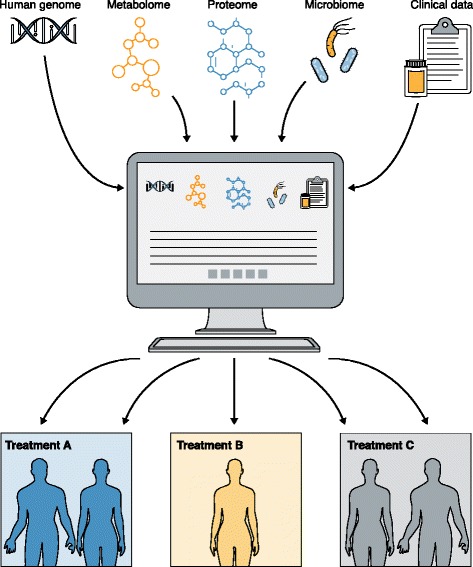
Clinical data are combined with ‘omics’ data sets, including microbiome analyses, in precision medicine strategies to identify personalized treatment options for individuals presenting with a given disease

A benefit of the microbiome in precision medicine is the ease of manipulation and delivery of therapeutics aimed at modulating microbiome functions. Continued research on the organisms and functions that affect disease and their variability among individuals is required to fully exploit the potential use of the microbiome in precision medicine. The adoption of routine sampling associated with wellness visits to a primary care physician, in combination with human genome and other clinical data, may reveal early signs of disease and may enhance the decision process around treatment options (Fig. 1). The interpretation of these composite data would be incomplete without a microbiome readout as, for example, the levels of many metabolites detected in metabolomic assays are influenced or produced by the microbiome. The interrelationships between the human genome, the microbiome, the metabolome, the proteome, the epigenome, the transcriptome, and other factors that provide a full picture of our health, are just starting to be revealed [10, 11]. While funding may not be immediately available to launch new studies in all these areas in health and disease, there is much to be gained by appropriately collecting and banking samples for future analyses, particularly from large cohort studies where integrative analyses of big data sets can be most meaningful.

## Abbreviations

IBD: Inflammatory bowel disease
PD-1: Programmed cell death protein 1
PD-L1: Programmed cell death 1 ligand 1
